# Trends in kidney stone disease: a 5‐year update of stone management in England and Scotland

**DOI:** 10.1111/bju.70409

**Published:** 2026-08-12

**Authors:** Catherine E. Lovegrove, Robert Geraghty, Imran Ahmad, Hing Y. Leung, Ben Turney

**Affiliations:** ^1^ School of Cancer Sciences University of Glasgow Glasgow UK; ^2^ Department of Urology University Hospital Monklands Airdrie UK; ^3^ Newcastle University Newcastle upon Tyne UK; ^4^ Department of Urology Freeman Hospital Newcastle upon Tyne UK; ^5^ Department of Urology Queen Elizabeth University Hospital Glasgow UK; ^6^ Nuffield Department of Surgical Sciences University of Oxford Oxford UK; ^7^ Department of Urology Oxford University Hospitals NHS Foundation Trust Oxford UK

**Keywords:** nephrolithiasis, urolithiasis, epidemiology, kidney stone disease, diagnosis, management

## Abstract

**Objectives:**

To describe contemporary trends in kidney stone disease (KSD)‐related inpatient episodes, procedures, and costs in England and Scotland, with emphasis on post‐coronavirus disease 2019 (COVID‐19) service recovery, socioeconomic variation in Scotland, comparison with other urological procedures, and 10‐year healthcare demand and cost projections.

**Materials and Methods:**

Retrospective descriptive analyses were conducted using English Hospital Episode Statistics (2020–2025) and Public Health Scotland data (2019–2024). KSD‐related finished consultant inpatient episodes and procedures were identified using the International Statistical Classification of Diseases and Related Health Problems 10th Revision and Operation and Procedures fourth edition codes, respectively. Procedures were categorised as extracorporeal shockwave lithotripsy, ureterorenoscopy (URS), percutaneous nephrolithotomy, or open. Trends were compared with uro‐oncological resections and benign prostate obstruction procedures. Socioeconomic variation was assessed using the Scottish Index of Multiple Deprivation. The 10‐year healthcare demand and cost projections, accounting for National Health Service (NHS) cost uplift factors, were modelled using historical data.

**Results:**

The COVID‐19 pandemic disproportionately reduced KSD‐associated activity compared with procedures for bladder and kidney cancer. Although volumes now exceed pre‐pandemic levels, inpatient episodes remain below baseline and waiting times remain prolonged. Between 2020 and 2025, inpatient episodes increased by 27.4% in England and 17% in Scotland, with URS accounting for >50% of interventions. KSD procedural volumes are comparable to the combined volume of uro‐oncological resections and benign prostatic obstruction procedures. In Scotland, 44% of procedures were performed in the two most deprived socioeconomic quintiles versus 37% in the two least deprived. In England, KSD‐associated episodes are projected to increase by 33% to 111 907 (95% confidence interval 96 057–130 373) by 2034–2035, with an estimated cumulative NHS cost of £1.82 billion over the next decade.

**Conclusion:**

Kidney stone disease exacts a growing burden on healthcare services with incomplete recovery of inpatient care following the COVID‐19 pandemic, socioeconomic disparities, and rising healthcare demand. These findings can inform service planning and prioritise evaluation of strategies to improve efficiency, equity, and prevention.

**Abbreviations**
COVID‐19: coronavirus disease 2019
eDRIS: Electronic Data Research and Innovation Service
ESWL: extracorporeal shockwave lithotripsy
HES: Hospital Episode Statistics
HRG: Healthcare Resource Group
KSD: kidney stone disease
PCNL: percutaneous nephrolithotomy
SMID: Scottish Index of Multiple Deprivation
URS: ureterorenoscopy

## Introduction

Kidney stone disease (KSD) has imposed an increasing burden on healthcare systems over the past 15 years, with rising incidence, diagnostic activity, and healthcare utilisation related to its management and prevention [1, 2, 3, 4]. Advances in endourological technology have improved procedural outcomes, increasing stone‐free rates through developments in extracorporeal shockwave lithotripsy (ESWL) [5], ureterorenoscopy (URS) [6, 7], and percutaneous nephrolithotomy (PCNL) [8, 9, 10]. However, little change has occurred in the medical management of KSD over the past two decades with the last real change being the introduction of potassium citrate in the 1980s [11, 12, 13, 14].

Kidney stone disease generates substantial and growing costs for healthcare systems [15]. In England, projections estimate that KSD will cost NHS England >£300 million annually by 2030, a financial burden comparable to the combined initial 5‐year management costs of prostate and bladder cancer [15]. KSD also affects the wider economy, as most patients are of working age and experience recurrent symptoms, hospital admissions, and procedural interventions that result in lost productivity and time away from work.

As demand for urological services continues to rise, healthcare providers must plan and allocate the workforce effectively to improve service efficiency, reduce waiting times, and maintain patient safety. National administrative datasets play a critical role in supporting this process. Hospital Episode Statistics (HES) provide comprehensive, curated data on hospital admissions and procedures across NHS hospitals in England and allow detailed assessment of historical trends to inform future service planning [16]. In Scotland, the Electronic Data Research and Innovation Service (eDRIS) links real‐world administrative datasets for research or health service audits and service improvement [17].

In this study, we describe 5‐year trends in inpatient hospital episodes and procedural interventions associated with KSD in England. We expand upon previous quintennial analyses and capture data from 2019 to 2025 to provide the first comprehensive evaluation of post‐coronavirus disease 2019 (COVID‐19) recovery of KSD services, incorporate the first national Scottish analysis using comparable methodology, evaluate socioeconomic variation in Scotland, compare KSD activity with contemporary uro‐oncological and benign prostate obstruction procedures, and update health‐economic projections using current NHS tariff data.

## Materials and Methods

### Data Aggregation

Methodology previously reported for quintennial analyses of Hospital Admitted Patient Care Activity Data [1, 3, 4, 16] were applied. The study periods represent the fourth 5‐year analysis of trends in procedural interventions for KSD in England and the first for Scotland. Given the potential impact of the COVID‐19 pandemic on healthcare activity in 2020–2021, we present data from 2019 to provide a context of pre‐pandemic activity. English data are aggregated from 1 April to 31 March the following year. Diagnoses of KSD were identified using International Classification of Diseases and Related Health Problems 10th Revision (ICD‐10) codes N20.0, N20.1, N20.2, N20.9, and N23.X (Table S1). The numbers of episodes were recorded as finished consultant episodes. Procedures were identified using the Office of Population Censuses and Surveys Classification of Interventions and Procedures, 4th edition (OPCS‐4) codes and grouped according to procedure type including ESWL, URS, PCNL, and open procedures (Table S1). We compared data with other index urology procedures, specifically nephrectomy, cystectomy, prostatectomy, and benign prostatic obstruction surgery including minimally invasive surgical techniques (Table S1).

We obtained data from 2019 to 2024 from eDRIS at Public Health Scotland using methods comparable to those applied by NHS England; however, Scottish data are aggregated by calendar year.

To evaluate the costs associated with procedures for KSD in England, we derived procedure‐specific cost estimates using the 2024–2024 NHS Payment Tariff [18] to reflect contemporaneous reimbursement rates for the final year of HES data available. For each procedure type, relevant Healthcare Resource Group (HRG) codes were identified to represent minimum, maximum, and most likely treatment costs (Table S2). The lower cost estimate assumed all patients received the least expensive HRG‐coded procedure, whereas the upper estimate assumed all patients received the most expensive HRG‐coded procedure. The most likely cost estimate was based on the HRG code judged to best represent typical clinical practice for the majority of urology patients in the UK, agreed by two authors.

### Future Projections

We subsequently projected costs related to KSD care over a 10‐year horizon, modelling variation based on trends in diagnoses in the last 27 years. First, we calculated the baseline ratio of total diagnoses (finished consultant episodes) to total procedures in England for 2024–2025 and assumed this to remain constant over the projection window. Second, the proportion of elective vs emergency procedures was similarly assumed to remain constant. Third, to project the absolute number of annual diagnoses, we fitted a generalised linear model with a quasi‐Poisson error distribution to trends spanning 1998–1999 to 2024–2025 and generated 95% confidence intervals (CI). Fourth, we used a generalised linear model with quasi‐binomial adjustment to project the changing proportions of ESWL, URS, PCNL, and open procedures relative to all procedures, using trends from 2000–2001 to 2024–2025.

To calculate the total annual projected cost of KSD‐related, the individual costs of the following clinical pathway components were aggregated:

A diagnostic CT scan for every projected diagnosis (£79, HRG code RD20A [19]).

- A preoperative clinic appointment for all projected elective operations (£165, HRG code WF01B [19]).
- ESWL, URS, PCNL, and open operations in their estimated annual proportions estimated, calculated using the ‘likely’ procedural tariffs (Table S2).
- A follow‐up CT scan and post‐procedural follow‐up appointment (£81, HRG code WF01A [19]) for every procedure, in accordance with the ‘Getting It Right First Time (GIRFT) Greener Pathways’ guide [20].

The total estimated cost for each future year was adjusted using a compound inflation multiplier based on the official NHS cost uplift factors (4.83% in 2025–2026, 2.03% in 2026–2027, and 3.0% per year thereafter) [21].

### Statistical Analyses

All data analysis was performed using RStudio, version 4.1 [22]. Descriptive data are reported as counts with percentages. To provide nominal budget forecasts, we fitted a generalised linear model (described above) and performed sensitivity analyses to provide upper and lower bounds for the projections; the lower boundary combined the lower 95% CI of projected diagnoses with ‘minimum’ HRG procedural costs, and the upper boundary combined the upper 95% CI for projected diagnoses with ‘maximum’ HRG procedural costs (Table S2), respectively.

### Ethics and Governance

The HES and Public Health Scotland data comprise anonymised routinely collected administrative datasets. Formal NHS Research Ethics Committee approval was not required as only aggregate, non‐identifiable data were analysed. Data were accessed in accordance with Public Health Scotland governance arrangements. To protect patient confidentiality, we censored demographic information for patients undergoing open procedures as counts were low, reducing the risk of inadvertent disclosure.

## Results

### Impact of the COVID‐19 Pandemic on KSD Activity

**Fig. 1:**
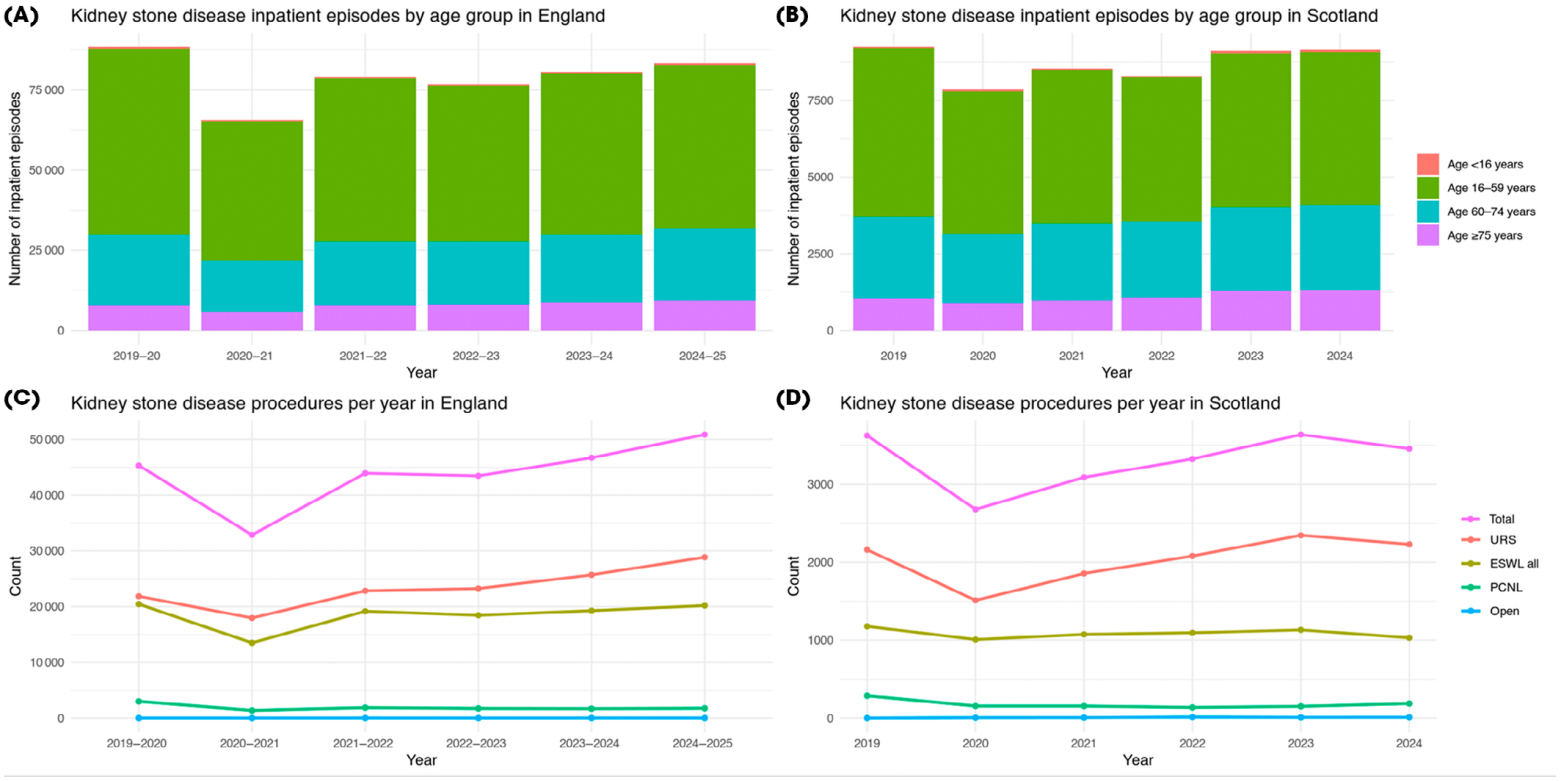
Number of inpatient episodes and procedures attributable to KSD in England and Scotland. Note different scales on *y*‐axes.

Between 2020 and 2021, the COVID‐19 pandemic was associated with a marked reduction in KSD‐related hospital episodes and procedural activity. In England, KSD episodes fell 25% below pre‐pandemic levels to 66 049 in 2020–2021 (88 632 episodes in 2019–2020). A 15% reduction was observed in KSD episodes in Scotland (9237 to 7849 episodes, Tables 1 and 2, Fig. 1A, B).

**Table 1:** Demographics of inpatient episodes and procedures for KSD in England.

Variable	2019–2020	2020–2021	2021–2022	2022–2023	2023–2024	2024–2025
**Episodes, *n* (%)**						
Total KSD episodes	88 632	66 049	79 412	77 152	81 083	84 130
Calculus of kidney (N20)	46 471 (52)	30 252 (46)	40 917 (52)	41 467 (54)	43 741 (54)	47 162 (56)
Calculus of ureter (N20.1)	26 904 (30)	23 757 (36)	25 584 (32)	24 102 (31)	25 504 (31)	25 010 (30)
Calculus of kidney and ureter (N20.2)	7629 (8.6)	6391 (10)	7321 (9.2)	6931 (9.0)	7483 (9.2)	7965 (9.5)
Urinary calculus, unspecified (N20.9)	605 (0.68)	596 (0.90)	566 (0.71)	546 (0.71)	577 (0.71)	567 (0.67)
Unspecified renal colic (N23.X)	7023 (7.9)	5053 (7.7)	5024 (6.3)	4106 (5.3)	3778 (4.7)	3426 (4.1)
Gender, *n* (%)						
Male	55 497 (63)	41 185 (62)	48 941 (62)	47 315 (61)	48 402 (60)	49 484 (59)
Female	33 123 (37)	24 860 (38)	30 464 (38)	29 832 (39)	31 851 (39)	33 991 (40)
Gender unknown	12 (0.014)	4 (0.01)	7 (0.01)	5 (0.01)	830 (1.0)	655 (0.78)
Age						
Age, years, mean	51.8	51.3	52.4	52.7	52.8	53.3
Age <16 years, *n* (%)	468 (0.53)	292 (0.44)	292 (0.37)	302 (0.39)	322 (0.40)	316 (0.38)
Age 16–59 years, *n* (%)	58 024 (65)	43 536 (66)	50 676 (64)	48 502 (63)	50 332 (62)	50 977 (61)
Age 60–74 years, *n* (%)	21 998 (25)	15 792 (24)	20 029 (25)	19 722 (26)	21 096 (26)	22 452 (27)
Age ≥75 years, *n* (%)	7844 (8.9)	5883 (8.9)	7804 (10)	8015 (10)	8663 (11)	9413 (11)
Age NA, *n* (%)	298 (0.34)	546 (0.83)	611 (0.77)	611 (0.79)	670 (0.83)	972 (1.2)
**Procedures, *n* (%)**						
Total KSD procedures	43 564	32 864	43 927	43 463	46 694	50 851
PCNL	2988 (6.6)	1379 (4.2)	1876 (4.3)	1731 (4.0)	1692 (3.6)	1765 (3.5)
URS	21 859 (48)	17 968 (55)	22 844 (52)	23 253 (54)	25 693 (55)	28 860 (57)
ESWL kidney	16 799 (37)	9294 (28)	14 519 (33)	14 148 (33)	14 841 (32)	15 929 (31)
ESWL ureter	3661 (8.1)	4203 (12.8)	4659 (10.6)	4309 (9.9)	4439 (9.5)	4268 (8.4)
ESWL all	20 460 (45)	13 497 (41)	19 178 (44)	18 457 (42)	19 280 (41)	20 197 (40)
Open	22 (0.05)	20 (0.06)	29 (0.07)	22 (0.05)	29 (0.06)	29 (0.06)
Gender, *n* (%)						
Male	29 233 (65)	21 227 (65)	27 823 (63)	27 657 (64)	28 861 (62)	31 352 (62)
Female	16 092 (36)	11 636 (35)	16 102 (37)	15 803 (36)	17 306 (37)	19 144 (38)
Gender unknown	4 (0.01)	1 (0.003)	2 (0.005)	3 (0.01)	527 (1.1)	355 (0.70)
Age						
Age, years, mean	54.2	53.9	54.4	54.5	54.5	54.7
Age < 16 years, *n* (%)	189 (0.42)	182 (0.55)	231 (0.53)	220 (0.51)	240 (0.51)	261 (0.51)
Age 16–59 years, *n* (%)	27 464 (61)	19 931 (61)	26 246 (60)	25 721 (59)	27 264 (58)	29 383 (58)
Age 60–74 years, *n* (%)	13 090 (29)	9297 (28)	12 702 (29)	12 607 (29)	13 802 (30)	15 041 (30)
Age ≥ 75 years, *n* (%)	4414 (9.7)	3229 (10)	4542 (10)	4663 (11)	5102 (11)	5695 (11)
Age NA, *n* (%)	172 (0.38)	225 (0.68)	206 (0.47)	252 (0.58)	286 (0.61)	471 (0.93)
Status, *n* (%)						
Emergency procedures*	2672 (5.9)	2421 (7.4)	2894 (6.6)	3026 (7.0)	3576 (7.7)	3403 (6.7)
Planned procedures*	7853 (17)	5014 (15)	6761 (15)	5475 (13)	6117 (13)	7087 (14)
Unknown emergency/planned	34 804 (77)	25 429 (77)	34 272 (78)	34 962 (80)	37 001 (79)	40 361 (79)
Bed days	33 615	22 888	30 244	28 896	32 799	33 424
Day case procedures, *n* (%)	29 238 (65)	21 028 (64)	29 162 (66)	29 903 (69)	32 291 (69)	35 439 (70)
Waiting time, days, median	47	42	43	49	53	53

* Data not available for all procedures.

**Table 2:** Demographics of inpatient episodes and procedures for KSD in Scotland.

Variable	2019	2020	2021	2022	2023	2024
**Episodes, *n* (%)**						
Total KSD episodes	9237	7849	8525	8304	9125	9167
Calculus of kidney (N20)	4243 (46)	3349 (43)	3691 (43)	3823 (46)	4193 (46)	4253 (46)
Calculus of ureter (N20.1)	3375 (37)	3131 (40)	3386 (40)	3197 (39)	3501 (38)	3508 (38)
Calculus of kidney and ureter (N20.2)	640 (6.9)	583 (7.4)	658 (7.7)	604 (7.3)	687 (7.5)	692 (7.5)
Urinary calculus, unspecified (N20.9)	170 (1.8)	156 (2.0)	154 (1.8)	112 (1.3)	98 (1.1)	90 (1.0)
Unspecified renal colic (N23.X)	809 (8.8)	630 (8.0)	636 (7.5)	568 (6.8)	645 (7.1)	624 (6.8)
Age, *n* (%)						
<16 years	37 (0.40)	49 (0.62)	37 (0.43)	31 (0.37)	99 (1.1)	91 (1.0)
16–59 years	5502 (60)	4642 (59)	5006 (59)	4704 (57)	4996 (55)	4984 (54)
60–74 years	2650 (29)	2266 (29)	2500 (29)	2505 (30)	2752 (30)	2783 (30)
≥75 years	1048 (11)	892 (11)	982 (12)	1064 (13)	1278 (14)	1309 (14)
**Procedures, *n* (%)**						
Total KSD procedures	3625	2678	3090	3325	3637	3456
PCNL	286 (7.9)	154 (5.8)	154 (5.0)	136 (4.1)	150 (3.1)	186 (5.4)
URS	2160 (60)	1511 (56)	1855 (60)	2080 (63)	2345 (49)	2229 (64)
ESWL kidney	716 (20)	479 (18)	588 (19)	714 (21)	689 (14)	649 (19)
ESWL ureter	462 (13)	528 (20)	486 (16)	381 (11)	443 (9.3)	381 (11)
ESWL all	1178 (32)	1007 (38)	1074 (35)	1095 (33)	1132 (24)	1030 (30)
Open	1 (0.03)	6 (0.2)	7 (0.2)	14 (0.4)	10 (0.2)	11 (0.3)
Gender, *n* (%)						
Female	1249 (35)	923 (26)	1116 (31)	1149 (32)	1299 (36)	1343 (37)
Male	2375 (66)	1749 (48)	1967 (54)	2162 (60)	2327 (64)	2102 (58)
Unknown	0 (0.00)	0 (0.00)	0 (0.00)	0 (0.00)	1 (0.03)	0 (0.00)
Age, *n* (%)						
<16 years	15 (0.4)	17 (0.6)	16 (0.5)	24 (0.7)	26 (0.7)	33 (1.0)
16–59 years	2080 (57)	1511 (56)	1737 (56)	1876 (56)	1937 (53)	1831 (53)
60–74 years	1174 (32)	895 (33)	1019 (33)	1085 (33)	1201 (33)	1151 (33)
≥75 years	356 (9.8)	255 (9.5)	318 (10)	342 (10)	473 (13)	441 (13)
SIMD, *n* (%)						
1	789 (22)	594 (22)	676 (22)	703 (21)	784 (22)	732 (21)
2	794 (22)	603 (23)	672 (22)	746 (22)	764 (21)	739 (21)
3	700 (19)	493 (18)	569 (18)	626 (19)	754 (21)	695 (20)
4	686 (19)	512 (19)	608 (20)	672 (20)	709 (19)	669 (19)
5	639 (18)	465 (17)	538 (17)	557 (17)	601 (17)	598 (17)
Unknown	17 (0.47)	11 (0.30)	27 (0.74)	21 (0.58)	25 (0.69)	23 (0.63)
Status, *n* (%)						
Emergency procedures	303 (8.4)	336 (13)	336 (11)	420 (13)	518 (14)	516 (15)
Planned procedures	3321 (92)	2336 (87)	2747 (89)	2891 (87)	3109 (85)	2929 (85)
Unknown emergency/planned	1 (0.03)	6 (0.22)	7 (0.23)	14 (0.42)	10 (0.27)	11 (0.32)

Activity declined across all KSD procedural modalities during this period. The most pronounced reduction was observed in ESWL, which decreased by ~34% in England and 15% in Scotland between 2019–2020 and 2020–2021 (Tables 1 and 2, Fig. 1C, D, Tables S3 and S4). Reductions were also observed in URS (18% reduction in England, 30% reduction in Scotland) and PCNL (54% reduction in England, 46% reduction in Scotland), reflecting widespread disruption to elective surgical services.

**Fig. 2:**
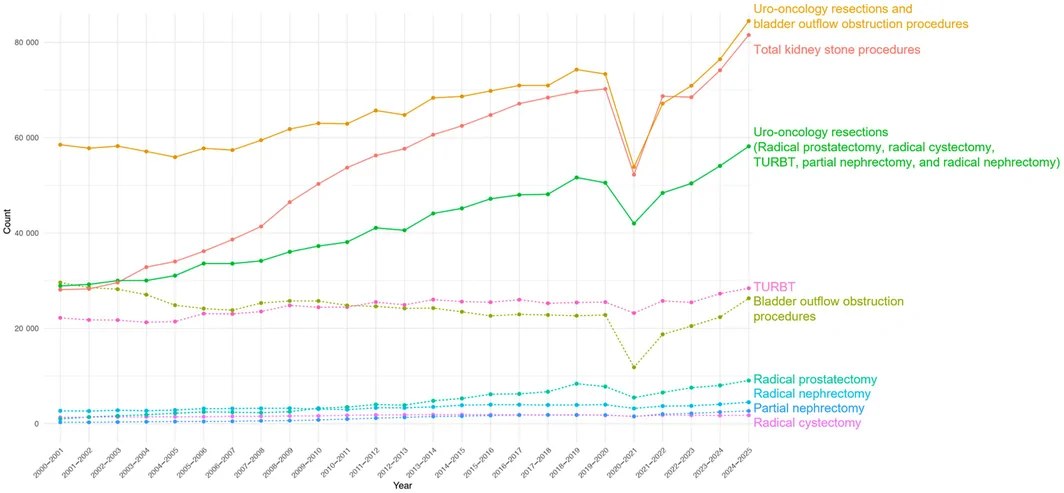
Number of procedures attributable to KSD in England compared to procedures for bladder outflow obstruction, kidney, bladder, and prostate cancer. ‘Total kidney stone procedures’ combine ESWL, open, PCNL, and URS procedures. ‘Uro‐oncology resections’ combine radical prostatectomy, radical nephrectomy, partial nephrectomy, transurethral resection of bladder tumour (TURBT), and radical cystectomy.

Analysis of HES data demonstrates that KSD procedural activity was disproportionately affected relative to other urological procedures (Fig. 2). Although KSD procedures had historically exceeded the combined volume of major oncological resections since 2003–2004, the pandemic period was associated with a relative contraction in stone‐related activity compared with oncological urological procedures (30% vs 14% reduction in activity, respectively; Fig. 2, Table S5).

### Recovery of KSD Services Following the Pandemic

Following the initial decline, KSD activity recovered progressively in both England and Scotland. By 2024–2025, KSD‐related hospital episodes in England had increased to 84 130 episodes, representing a 27.4% rise from the pandemic nadir, although remaining 5.08% below pre‐pandemic levels when compared with 2019–2020 data (Table 1, Fig. 1A).

A similar recovery pattern was observed in Scotland, where overall activity remained slightly below pre‐pandemic levels (−0.76%) when analyses included 2019 data (Table 2).

Procedural volumes demonstrated a comparable recovery trajectory, with sustained growth particularly in URS (Tables 1 and 2, Fig. 1C, D). However, despite recovery in activity, waiting times—especially for URS and PCNL—remained prolonged compared with pre‐pandemic levels (Table 1).

By 2024–2025, KSD procedural volume in England had recovered to a level nearly equivalent to the combined total of oncological resections and benign prostatic obstruction procedures, although the gap observed pre‐pandemic had narrowed, largely reflecting recovery in benign prostatic obstruction procedures and radical prostatectomy (Fig. 2, Table S5).

### Current Demographic Trends in KSD

The mean age of patients admitted with KSD in England during the study period was 52.5 years, representing an increase of 2.5 years compared with the preceding 5‐year period and demonstrating a consistent year‐on‐year rise (Table 1). Although the largest proportion of KSD episodes continues to occur in patients aged 16–59 years, the proportion attributable to older age groups (60–74 years and ≥75 years) has increased over time in both England and Scotland (Tables 1 and 2). In England, paediatric KSD episodes have declined compared with the preceding 5‐year period (annual average 2015–2019: 492 episodes vs 305 episodes in 2020–2025), a trend not observed in Scotland (Table 1, Fig. 1A, B).

Scottish data demonstrate a persistent socioeconomic gradient in procedural utilisation, with a higher proportion of procedures performed in more deprived groups (Scottish Index of Multiple Deprivation [SIMD] 1–2) and lower proportions in the least deprived group (SMID 5), with minimal change over time (Table 2).

### Contemporary Procedural Patterns

Ureterorenoscopy remains the most frequently performed intervention in both nations, accounting for >50% of all KSD procedures. In England, annual URS activity increased from 22 844 procedures in 2020–2021 to 28 860 in 2024–2025 (26.3% increase), with a more pronounced rise observed in Scotland (47.1% increase) (Fig. 1C, D, Tables S3 and S4).

Median waiting times for URS in England increased from approximately 6.5 weeks to 8 weeks over the study period, despite a 14% rise in day‐case procedures, with more than half now performed as day cases (Table S3).

Extracorporeal shockwave lithotripsy activity has declined overall. In England, the total number of ESWL procedures has decreased modestly by 1.3% compared with 2019–2020 levels (20 460 procedures in 2019–2020; 20 197 in 2024–2025, Table S3, Fig. 1C). Scottish data demonstrate a larger overall reduction in ESWL activity of 15.3% between 2019 and 2024 (Table S4). In both nations, ESWL has accounted for a progressively smaller proportion of total KSD. Median waiting times for ESWL in England exceeded 1 month in 2023–24, and >90% of ESWL procedures are performed as day cases (Table S3, Fig. 1D).

Percutaneous nephrolithotomy accounts for a decreasing proportion of KSD interventions in both England (4.2% of procedures in 2020–2021 and 3.5% of procedures in 2024–2025) and Scotland (7.9% of procedures in 2020 and 5.4% of procedures in 2025; Fig. 1C, D, Tables S3 and S4). Median waiting times for PCNL in England ranged from 73 to 91 days.

Open surgical interventions remain rare, accounting for <1% of all KSD procedures, consistent with previously reported trends (Tables S3 and S4, Fig. 1C, D).

### The 10‐Year Healthcare Demand and Budget Projections

#### Model Fit

Historical HES data spanning 1998–1999 to 2024–2025 demonstrated an increase in total KSD diagnoses (*P* < 0.001, Table S6). The quasi‐Poisson model demonstrated an excellent fit to the historical data (residual deviance: 31070 on 26 degrees of freedom). From 2000–2001 to 2024–2025, there were significant changes with decreases in the proportion of ESWL for renal stones, open procedures, and increases in URS (each model *P* < 0.001, Table S6). The relative proportion of ESWL for ureteric stones and PCNL remained unchanged (*P* = 0.88 and *P* = 0.31, respectively).

#### Projections

In 2024–2025, the ratio of total procedures to diagnoses was 0.60 (50 851 and 84 130, respectively, Table 1, Table S6). Maintaining this ratio, the absolute volume of KSD‐associated consultant episodes in England is projected to reach 111 907 (95% CI 96 057–130 373) by 2034–2035 representing a 33% increase from 2024 to 2025 (95% CI 14–55%, Table S7). Driven by this rise, we project an annual total of 81 731 procedures for KSD in 2034–2035 (95% CI 70 154–95 216) with URS accounting for >67% of cases and PCNL for <5% (Table S7).

**Fig. 3:**
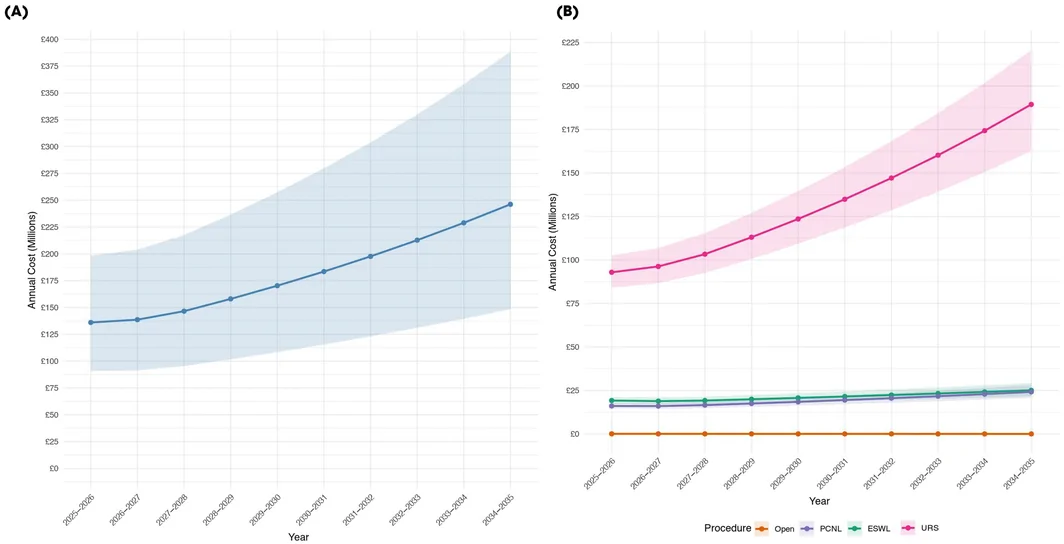
Projected annual cost of treating KSD in England from 2025 to 2035. (**A**) Annual cost of managing all kidney stone episodes; (**B**) Annual cost of procedures for KSD. Shaded areas represent upper and lower 95% CIs. Projections are estimated from episode volume (1998–1999 to 2024–2025) and procedural trends (2000–2001 to 2024–2025) and account for NHS cost uplift factors.

Using the ‘likely’ HRG tariff costs and incorporating routine clinical pathway components (diagnostic imaging, post‐procedural imaging, pre‐ and postoperative appointments), the total nominal cost of KSD care in England is projected to rise to £246 million by 2034–2035 (Table 1) and the cumulative nominal cost to the NHS over the 10‐year projection window (2025–2026 to 2034–2035) is estimated at £1.82 billion under the primary scenario (Table S8, Fig. 3). In a conservative sensitivity scenario, the projected annual cost by 2034–2035 is estimated at £149 million. Conversely, under the upper boundary scenario, annual costs are projected to reach £389 million by 2034–2035 (Table S8, Fig. 3).

## Discussion

These analyses represent the fourth assessment of NHS activity related to KSD in England, the first in Scotland, and provide a comprehensive UK‐wide evaluation not only of the impact of the COVID‐19 pandemic on KSD‐related activity, but also the recovery from it. Our findings demonstrate that KSD services were disproportionately disrupted, compared with other urological procedures, and that the recovery period features procedural activity exceeding pre‐pandemic levels but incomplete restoration of inpatient episodes and prolonged waiting times.

It is well‐documented that the COVID‐19 pandemic substantially reduced both inpatient episodes and procedural volumes across surgical specialties [23, 24, 25, 26]. However, in this study we identify that KSD services were disproportionately affected, with greater reduction in KSD‐related procedural activity than that observed for oncological urological interventions, suggesting that patients with KSD experienced greater disruption to care during periods of constrained elective capacity. Furthermore, while procedural activity recovered by 2023–2024 and now exceed pre‐pandemic levels (Fig. 2, Table S5), inpatient episodes remain below baseline (Fig. 1) and waiting times—particularly for URS and PCNL—remain prolonged. These findings intimate that the recovery of KSD services remains incomplete several years after the pandemic, intimating persistent changes in patterns of care delivery rather than full system normalisation, an observation not captured on earlier reports that focus on the acute effects of the pandemic [27, 28, 29, 30, 31, 32].

Kidney stone disease continues to predominantly affect individuals of working age (Tables 1 and 2). However, the increasing proportion of older patients may have important implications for future workforce and service planning, as this population is associated with multimorbidity, increased perioperative complexity, and greater healthcare resource utilisation, including longer hospital stays and more complex discharge pathways [33]. Previously unexplored Scottish data demonstrate a persistent socioeconomic gradient in procedural utilisation, with higher proportions of interventions performed in more deprived populations (Table 2). Although overall trends in procedural activity were broadly comparable with England, the Scottish dataset provides additional insight by enabling assessment of socioeconomic variation within a national healthcare system with universal access. Previous studies report associations of lower socioeconomic status with greater KSD burden and more complex stone disease [34, 35]. The observed gradient may reflect multiple factors, including variation in disease prevalence, multimorbidity, patterns of presentation, and healthcare utilisation. While our analysis cannot determine the relative contribution of these factors, these findings highlight the importance of incorporating socioeconomic considerations into future KSD service planning. Targeted prevention, optimisation of modifiable risk factors, and equitable resource allocation may help ensure that evolving models of care do not inadvertently widen existing health inequalities.

Health economic analyses demonstrate a substantial and sustained rise in costs associated with KSD management and may have broader policy and public health implications. As KSD disproportionately affects the working‐age population (Tables 1 and 2), it represents both a healthcare and economic challenge. Previous projections suggest that overall NHS England expenditure on KSD will exceed £300 million annually by 2030 [15]. Our findings support this trajectory with an estimated 10‐year cumulative cost of greater than £1.82 billion. While our analyses include procedural tariffs alone, and do not incorporate the cost of primary care attendance, elective/emergency inpatient admissions, metabolic evaluation, prescriptions, repeat attendances, administrative, and indirect societal costs, they likely underestimate the true economic burden when wider resource utilisation is included. Furthermore, we acknowledge that the next 10 years will see changes in patient demographics, technology, and health budget restrictions that we have not accounted for, and which will affect patient outcomes.

Rising procedural volumes and projected costs provide evidence of increasing demand that may inform future service planning. With KSD procedures in England approaching volumes comparable to the combined volume of uro‐oncological resections and benign prostate obstruction procedures, these findings highlight the importance of ensuring that theatre capacity, consumables, workforce, and infrastructure are aligned with population need. However, further research is required to determine which service interventions, including technological advances and artificial intelligence, are most effective in improving efficiency and reducing waiting times [36]. Potential areas to evaluate include pathway optimisation, ambulatory care, preoperative assessment strategies, theatre efficiency and day‐case pathways, and approaches to preventing the incidence and recurrence of KSD that may help manage future demand while maintaining quality of care.

This study has several limitations. Both HES and Public Health Scotland data rely on accurate clinical coding. Misclassification or incomplete coding may lead to underestimation of procedural activity, which may explain the apparent decrease in KSD procedures in Scotland in 2024, meaning the true burden of KSD is likely even higher. The aggregate data cannot provide the granularity to identify *de novo* vs recurrent presentations. However, our analyses represent the current demands of KSD care on NHS services in England and Scotland regardless of whether a patient is a known kidney stone former. In economic analyses, procedures may attract multiple possible tariffs; while we considered minimum, maximum, and most likely costs, agreed by two authors, these estimates are subjective.

## Conclusions

In conclusion, KSD continues to place an increasing burden on NHS services in England and Scotland, with rising procedural demand, increasing healthcare costs, and prevalence of disease in patients of working age and from lower socioeconomic status. Although procedural activity has recovered following the COVID‐19 pandemic, inpatient activity and waiting times suggest that recovery remains incomplete several years later. These contemporary national data provide evidence to inform future workforce and service planning, identify socioeconomic inequalities that warrant further investigation, and quantify the projected economic burden of KSD. Future studies should evaluate which service innovations and preventive strategies are most effective in improving efficiency, reducing inequalities, and meeting the growing demand for KSD care.

## Disclosure of Interests

No conflicts exist.

## Supporting information


**Table S1.** Inclusion codes for diagnoses and procedures.
**Table S2.** The 2024–2025 NHS tariff costs used as baseline in healthcare economic analyses.
**Table S3.** Demographics of inpatient episodes and procedures for KSD in England stratified by procedure type.
**Table S4.** Demographics of inpatient episodes and procedures for KSD in Scotland stratified by procedure type.
**Table S5.** Comparison of annual activity in England.
**Table S6.** Historic trends in diagnoses and procedures.
**Table S7.** Projected procedural expenditure on KSD from 2025 to 2035.
**Table S8.** Projected procedural expenditure on KSD from 2025 to 2035.

## Data Availability

The data that supports the findings of this study are available in the Supporting Information of this article.
