# Supplementary material for: Trends in kidney stone disease: a 5‐year update of stone management in England and Scotland
Source: BJU Int. 2026 Aug 12;138(4):620–9. doi: 10.1111/bju.70409 (PMC13550797; doi:10.1111/bju.70409)
Supplement: Supplementary file 1 [file BJU-138-620-s001.docx]

Supplementary table 1. Inclusion codes for diagnoses and procedures

| **Category** | **Code** | **Description** |
| --- | --- | --- |
| Diagnosis | ICD10-N20.0 | Calculus of kidney |
|  | ICD10-N20.1 | Calculus of ureter |
|  | ICD10-N20.2 | Calculus of kidney with calculus of ureter |
|  | ICD10-N20.9 | Urinary calculus, unspecified |
|  | ICD10-N23.X | Unspecified renal colic |
| URS | OPCS4- M07.1 | Ureteroscopic laser fragmentation of calculus of kidney |
|  | OPCS4- M07.2 | Ureteroscopic extraction of calculus of kidney NEC |
|  | OPCS4- M09.1 | Endoscopic ultrasound fragmentation of calculus of kidney |
|  | OPCS4- M09.2 | Endoscopic electrohydraulic shockwave fragmentation of calculus of kidney |
|  | OPCS4- M09.3 | Endoscopic laser fragmentation of calculus of kidney |
|  | OPCS4- M09.8 | Endoscopic laser fragmentation of calculus of kidney -Other specified |
|  | OPCS4- M09.9 | Endoscopic laser fragmentation of calculus of kidney -Unspecified |
|  | OPCS4- M27.1 | Ureteroscopic laser fragmentation of calculus of ureter |
|  | OPCS4- M27.2 | Ureteroscopic fragmentation of calculus of ureter NEC |
|  | OPCS4- M27.3 | Ureteroscopic extraction of calculus of ureter |
|  | OPCS4- M28·1 | Endoscopic laser fragmentation of calculus of ureter |
|  | OPCS4- M28·2 | Endoscopic fragmentation of calculus of ureter |
|  | OPCS4- M28·3 | Endoscopic extraction of calculus of ureter |
|  | OPCS4- M28.4 | Endoscopic catheter drainage of calculus of ureter |
|  | OPCS4- M28.5 | Endoscopic drainage of calculus of ureter by dilation of ureter |
|  | OPCS4- M28.8 | Other endoscopic removal of calculus from ureter -Other specified |
|  | OPCS4- M28.9 | Other endoscopic removal of calculus from ureter -Unspecified |
| PCNL | OPCS4- M09.4 | Endoscopic extraction of calculus of kidney NEC |
|  | OPCS4- M16.4 | Percutaneous nephrolithotomy NEC |
|  | OPCS4- M26.1 | Nephroscopic laser fragmentation of calculus of ureter |
|  | OPCS4- M26.2 | Nephroscopic fragmentation of calculus of ureter NEC |
|  | OPCS4- M26.3 | Nephroscopic extraction of calculus of ureter |
| ESWL-kidney | OPCS4- M14.1 | Extracorporeal shock wave lithotripsy of calculus of kidney |
|  | OPCS4- M14.8 | Extracorporeal fragmentation of calculus of kidney -Other specified |
|  | OPCS4- M14.9 | Extracorporeal fragmentation of calculus of kidney- Unspecified |
| ESWL-ureter | OPCS4- M31.1 | Extracorporeal shockwave lithotripsy of calculus of ureter |
|  | OPCS4- M31.8 | Extracorporeal fragmentation of calculus of ureter -Other specified |
|  | OPCS4- M31.9 | Extracorporeal fragmentation of calculus of ureter -Unspecified |
| Open | OPCS4- M06.1 | Open removal of calculus from kidney |
| Radical nephrectomy | OPCS4- M02.1 | Total Excision Of Kidney, Nephrectomy And Excision Of Perirenal Tissue |
|  | OPCS4- M02.2 | Total Excision Of Kidney, Nephroureterectomy Nec |
|  | OPCS4- M02.3 | Total Excision Of Kidney, Bilateral Nephrectomy |
|  | OPCS4- M02.8 | Total Excision Of Kidney, Other Specified |
|  | OPCS4- M02.9 | Total Excision Of Kidney, Unspecified |
| Partial nephrectomy | OPCS4- M03.1 | Heminephrectomy of duplex kidney |
|  | OPCS4- M03.8 | M03.8 Other specified partial excision of kidney |
|  | OPCS4- M03.9 | M03.9 Unspecified partial excision of kidney |
|  | OPCS4- M02.4 | M02.4 Total Excision Of Kidney, Excision Of Half Of Horseshoe Kidney |
| Radical cystectomy | OPCS4- M34.1 | Total excision of bladder, Cystoprostatectomy |
|  | OPCS4- M34.2 | Total excision of bladder, Cystourethrectomy |
|  | OPCS4- M34.3 | Total excision of bladder, Cystectomy nec |
|  | OPCS4- M34.8 | Other specified total excision of bladder |
|  | OPCS4- M34.9 | Unspecified total excision of bladder |
| TURBT | OPCS4- M42.1 | Endoscopic resection of lesion of bladder |
|  | OPCS4- M42.3 | Endoscopic destruction of lesion of bladder NEC |
|  | OPCS4- M42.8 | Other specified endoscopic extirpation of lesion of bladder |
|  | OPCS4- M42.9 | Unspecified endoscopic extirpation of lesion of bladder |
| Radical prostatectomy | OPCS4- M61.1 | Total excision of prostate and capsule of prostate |
| Bladder outflow procedures | OPCS4- M65.1 | Endoscopic resection of prostate using electrotome |
|  | OPCS4- M65.2 | Endoscopic resection of prostate using punch |
|  | OPCS4- M65.3 | Endoscopic resection of prostate NEC |
|  | OPCS4- M65.4 | Endoscopic resection of prostate using laser |
|  | OPCS4- M65.5 | Endoscopic resection of prostate using vapotrode |
|  | OPCS4- M65.6 | Endoscopic ablation of prostate using steam |
|  | OPCS4- M65.8 | Other specified |
|  | OPCS4- M65.9 | Unspecified |
|  | OPCS4- M68.1 | Endoscopic insertion of prostatic stent |
|  | OPCS4- M68.2 | Endoscopic removal of prostatic stent |
|  | OPCS4- M68.3 | Endoscopic insertion of prosthesis to compress lobe of prostate |
|  | OPCS4- M68.8 | Other specified endoscopic insertion of prosthesis into prostate |
|  | OPCS4- M68.9 | Unspecified endoscopic insertion of prosthesis into prostate |

ESWL= extracorporeal shockwave lithotripsy; PCNL= percutaneous nephrolithotomy; TURBT= Transurethral resection of bladder tumour; URS= ureterorenoscopy

Supplementary table 2. 2024-2025 NHS Tariff costs used as baseline in healthcare economic analyses

| **Category** | **Strata** | **HRG code** | **Name** | Cost (£) |
| --- | --- | --- | --- | --- |
| ESWL | Min | LB36Z | Extracorporeal Lithotripsy | 496 |
|  | Max | LB36Z | Extracorporeal Lithotripsy | 496 |
|  | Likely | LB36Z | Extracorporeal Lithotripsy | 496 |
| Open | Min | LB61G | Major Open or Percutaneous | 5862 |
|  | Max | LB61C | Major Open or Percutaneous | 12058 |
|  | Likely | LB61F | Major Open or Percutaneous | 6406 |
| PCNL | Min | LB75B | Percutaneous Nephrolithotomy with CC Score 0-1 | 5071 |
|  | Max | LB75A | Percutaneous Nephrolithotomy with CC Score 2+ | 5948 |
|  | Likely | LB75B | Percutaneous Nephrolithotomy with CC Score 0-1 | 5071 |
| URS | Min | LB09D | Intermediate Endoscopic Ureter Procedures 19 years and over | 1313 |
|  | Max | LB64C | Complex Endoscopic Kidney or Ureter Procedures | 3561 |
|  | Likely | LB65E | Major Endoscopic Kidney or Ureter Procedures | 2364 |

CC= Charlson Comorbidity; ESWL= extracorporeal shockwave lithotripsy; HRG= Healthcare Resource Group; PCNL= percutaneous nephrolithotomy; TURBT= Transurethral resection of bladder tumour; URS= ureterorenoscopy

Supplementary table 3. Demographics of inpatient episodes and procedures for kidney stone disease in England stratified by procedure type

|  | | 2019-20 | 2020-21 | 2021-22 | 2022-23 | 2023-24 | 2024-25 |
| --- | --- | --- | --- | --- | --- | --- | --- |
| URS | | | | | | | |
| Total procedures | | 21859 | 17968 | 22844 | 23253 | 25693 | 28860 |
| Gender | Male | 13787 (63%) | 11306 (63%) | 14079 (62%) | 14284 (61%) | 15479 (60%) | 17232 (60%) |
|  | Female | 8070 (37%) | 6662 (37%) | 8763 (38%) | 8966 (39%) | 9981 (39%) | 11439 (40%) |
|  | Gender unknown | 2 (0.01%) | 0 (0.00%) | 2 (0.01%) | 3 (0.01%) | 233 (0.91%) | 189 (0.65%) |
| Age | Mean age (years) | 55 | 55 | 56 | 56 | 56 | 56 |
|  | Age <16 years | 85 (0.39%) | 76 (0.42%) | 111 (0.49%) | 118 (0.51%) | 116 (0.45%) | 117 (0.41%) |
|  | Age 16-59 years | 12475 (57%) | 10340 (58%) | 12976 (57%) | 13043 (56%) | 14324 (56%) | 15809 (55%) |
|  | Age 60-74 years | 6605 (30%) | 5254 (29%) | 6635 (29%) | 6910 (30%) | 7664 (30%) | 8848 (31%) |
|  | Age ≥75 years | 2590 (12%) | 2151 (12%) | 2976 (13%) | 3023 (13%) | 3436 (13%) | 3880 (13%) |
|  | Age NA | 104 (0.48%) | 147 (0.82%) | 146 (0.64%) | 159 (0.68%) | 153 (0.60%) | 206 (0.71%) |
| Status | Emergency* | 2272 (10%) | 2078 (12%) | 2078 (9.1%) | 2661 (11%) | 3175 (12%) | 3034 (11%) |
|  | Planned* | 1401 (6.4%) | 876 (4.9%) | 876 (3.8%) | 1019 (4.4%) | 1231 (4.8%) | 1804 (6.3%) |
| Bed days | | 24671 | 18296 | 18296 | 22955 | 27165 | 26054 |
| Day case | | 9172 (42%) | 7996 (45%) | 7996 (35%) | 11918 (51%) | 13585 (53%) | 16409 (57%) |
| Median waiting time (days) | | 49 | 45 | 45 | 49 | 54 | 59 |
| ESWL (kidney and ureter) | | | | | | | |
| Total procedures | | 20460 | 13497 | 19178 | 18457 | 19280 | 20197 |
| Gender | Male | 13807 (67%) | 9162 (68%) | 12730 (66%) | 12463 (68%) | 12509 (65%) | 13173 (65%) |
|  | Female | 6651 (33%) | 4334 (32%) | 6448 (34%) | 5994 (32%) | 6498 (34%) | 6879 (34%) |
|  | Gender unknown | 2 (0.01%) | 1 (0.01%) | 0 (0%) | 0 (0%) | 273 (1.4%) | 145 (0.72%) |
| Age | Mean age (years) | 53 | 52 | 53 | 53 | 53 | 53 |
|  | Age <16 years | 51 (0.25%) | 58 (0.43%) | 88 (0.46%) | 66 (0.36%) | 85 (0.44%) | 105 (0.52%) |
|  | Age 16-59 years | 13261 (65%) | 8826 (65%) | 12221 (64%) | 11713 (63%) | 12053 (63%) | 12642 (63%) |
|  | Age 60-74 years | 5609 (27%) | 3624 (27%) | 5463 (28%) | 5165 (28%) | 5584 (29%) | 5610 (28%) |
|  | Age ≥75 years | 1485 (7.3%) | 922 (6.8%) | 1356 (7.1%) | 1437 (7.8%) | 1436 (7.4%) | 1597 (7.9%) |
|  | Age NA | 54 (0.26%) | 67 (0.50%) | 50 (0.26%) | 76 (0.41%) | 122 (0.63%) | 243 (1.2%) |
| Status | Emergency* | 317 (1.5%) | 301 (2.2%) | 290 (1.5%) | 316 (1.7%) | 358 (1.9%) | 314 (1.6%) |
|  | Planned* | 6273 (31%) | 4075 (30%) | 5646 (29%) | 4394 (24%) | 4812 (25%) | 5209 (26%) |
| Bed days | | 1318 (6.4%) | 1199 (8.9%) | 1647 (8.6%) | 1663 (9.0%) | 1279 (6.6%) | 2906 (14%) |
| Day case | | 19515 (95%) | 12797 (95%) | 18403 (96%) | 17753 (96%) | 18521 (96%) | 18826 (93%) |
| Median waiting time (days) | | 33 | 22 | 28 | 29 | 39 | 36 |
| PCNL | | | | | | | |
| Total procedures | | 2988 | 1379 | 1876 | 1731 | 1692 | 1765 |
| Gender | Male | 1625 (54.4%) | 749 (54%) | 997 (53%) | 898 (52%) | 861 (51%) | 929 (53%) |
|  | Female | 1363 (46%) | 630 (46%) | 879 (47%) | 833 (48%) | 811 (48%) | 815 (46%) |
|  | Gender unknown | 0 (0%) | 0 (0%) | 0 (0%) | 0 (0%) | 20 (1%) | 21 (1.2%) |
| Age | Mean age (years) | 54 | 54 | 55 | 54 | 55 | 55 |
|  | Age <16 years | 52  (1.7%) | 44 (3.2%) | 29 (1.5%) | 35 (2.0%) | 37 (2.2%) | 37 (2.1%) |
|  | Age 16-59 years | 1716 (57%) | 759 (55%) | 1036 (55%) | 953 (55%) | 867 (51%) | 917 (52%) |
|  | Age 60-74 years | 871 (29%) | 410 (30%) | 596 (32%) | 525 (30%) | 550 (33%) | 576 (33%) |
|  | Age ≥75 years | 336 (11%) | 155 (11%) | 205 (11%) | 201 (12%) | 227 (13%) | 213 (12%) |
|  | Age NA | 13 (0.44%) | 11 (0.80%) | 10 (0.53%) | 17 (0.98%) | 11 (0.65%) | 22 (1.2%) |
| Status | Emergency* | 82 (2.7%) | 42 (3.0%) | 65 (3.5%) | 49 (2.8%) | 40 (2.4%) | 55 (3.1%) |
|  | Planned* | 176 (5.9%) | 62 (4.5%) | 68 (3.6%) | 62 (3.6%) | 72 (4.3%) | 74 (4.2%) |
| Bed days | | 1599 | 3318 | 4869 | 4210 | 4195 | 4386 |
| Day case | | 551 (18.4%) | 235 (17%) | 222 (12%) | 231 (13%) | 185 (11%) | 203 (12%) |
| Median waiting time (days) | | 73 | 82 | 91 | 83 | 91 | 86 |
| Open | | | | | | | |
| Total procedures | | 22 | 20 | 29 | 22 | 29 | 29 |
| Gender | Male | 14 (64%) | 10 (50%) | 17 (59%) | 12 (55%) | 12 (41%) | 18 (62%) |
|  | Female | 8 (36%) | 10 (50%) | 12 (41%) | 10 (45%) | 16 (55%) | 11 (38%) |
|  | Gender unknown | 0 (0.00%) | 0 (0.00%) | 0 (0%) | 0 (0%) | 1 (3.4%) | 0 (0.00%) |
| Age | Mean age (years) | 52 | 47 | 55 | 51 | 47 | 52 |
|  | Age <16 years | 1 (4.5%) | 4 (20%) | 3 (10%) | 1 (4.5%) | 2 (6.9%) | 2 (6.9%) |
|  | Age 16-59 years | 12 (55%) | 6 (30%) | 13 (45%) | 12 (55%) | 20 (69%) | 15 (52%) |
|  | Age 60-74 years | 5 (23%) | 9 (45%) | 8 (28%) | 7 (32%) | 4 (14%) | 7 (24%) |
|  | Age ≥75 years | 3 (14%) | 1 (5.0%) | 5 (17.2%) | 2 (9.1%) | 3 (10.3%) | 5 (17.2%) |
|  | Age NA | 1 (4.55%) | 0 (0.00%) | 0 (0.00%) | 0 (0.00%) | 0 (0.00%) | 0 (0.0%) |
| Status | Emergency* | 1 (4.5%) | 0 (0.0%) | 1 (3.4%) | 0 (0.0%) | 3 (10%) | 0 (0.0%) |
|  | Planned* | 3 (14%) | 1 (5.0%) | 1 (3.4%) | 0 (0%) | 2 (6.9%) | 0 (0%) |
| Bed days | | 95 | 75 | 84 | 68 | 160 | 78 |
| Day case | | 0 (0%) | 0 (0%) | 3 (10%) | 1 (5%) | 0 (0%) | 1 (3%) |
| Median waiting time (days) | | 62 | 56 | 79 | 118 | 121 | 81 |

ESWL= extracorporeal shockwave lithotripsy; PCNL= percutaneous nephrolithotomy; URS= ureterorenoscopy *data not available for all procedures

Supplementary table 4. Demographics of inpatient episodes and procedures for kidney stone disease in Scotland stratified by procedure type

|  | | 2019 | 2020 | 2021 | 2022 | 2023 | 2024 |
| --- | --- | --- | --- | --- | --- | --- | --- |
| URS | | | | | | | |
| Total procedures | | 2160 | 1511 | 1855 | 2078 | 2342 | 2222 |
| Gender | Male | 1375 (64%) | 952 (63%) | 1130 (61%) | 1319 (63%) | 1485 (63%) | 1362 (61%) |
|  | Female | 785 (36%) | 559 (37%) | 725 (39%) | 759 (37%) | 857 (37%) | 860 (39%) |
|  | Gender unknown | 0 (0%) | 0 (0%) | 0 (0%) | 0 (0%) | 0 (0%) | 0 (0%) |
| Age | Age <16 years | 5 (0.23%) | 0 (0%) | 0 (0%) | 0 (0%) | 0 (0%) | 0 (0%) |
|  | Age 16-59 years | 1206 (56%) | 805 (53%) | 983 (53%) | 1149 (55%) | 1201 (51%) | 1163 (52%) |
|  | Age 60-74 years | 701 (32%) | 527 (35%) | 626 (34%) | 675 (32%) | 800 (34%) | 750 (34%) |
|  | Age ≥75 years | 248 (11%) | 175 (12%) | 244 (13%) | 253 (12%) | 340 (15%) | 305 (14%) |
| Status | Emergency | 266 (12%) | 287 (19%) | 310 (17%) | 378 (18%) | 470 (20%) | 469 (21%) |
|  | Planned | 1894 (88%) | 1224 (81%) | 1545 (83%) | 1700 (82%) | 1872 (80%) | 1753 (79%) |
| ESWL (kidney and ureter) | | | | | | | |
| Total procedures | | 1178 | 1006 | 1074 | 1095 | 1130 | 998 |
| Gender | Male | 830 (70%) | 713 (71%) | 749 (70%) | 769 (70%) | 767 (68%) | 620 (62%) |
|  | Female | 348 (30%) | 293 (29%) | 325 (30%) | 326 (30%) | 363 (32%) | 378 (38%) |
|  | Gender unknown | 0 (0%) | 0 (0%) | 0 (0%) | 0 (0%) | 0 (0%) | 0 (0%) |
| Age | Age <16 years | 0 (0%) | 5 (0.50%) | 5 (0.47%) | 0 (0%) | 12 (1.1%) | 12 (1.2%) |
|  | Age 16-59 years | 729 (62%) | 619 (62%) | 672 (63%) | 652 (60%) | 650 (58%) | 559 (56%) |
|  | Age 60-74 years | 371 (31%) | 319 (32%) | 337 (31%) | 367 (34%) | 353 (31%) | 328 (33%) |
|  | Age ≥75 years | 75 (6.4%) | 63 (6%) | 60 (6%) | 72 (7%) | 115 (10%) | 98 (10%) |
| Status | Emergency | 25 (2.1%) | 44 (4.4%) | 17 (1.6%) | 35 (3.2%) | 43 (3.8%) | 39 (3.9%) |
|  | Planned | 1153 (98%) | 962 (96%) | 1057 (98%) | 1060 (97%) | 1087 (96%) | 959 (96%) |
| PCNL | | | | | | | |
| Total procedures | | 286 | 154 | 154 | 136 | 150 | 186 |
| Gender | Male | 170 (59%) | 84 (55%) | 88 (57%) | 72 (53%) | 73 (49%) | 95 (51%) |
|  | Female | 116 (41%) | 70 (45%) | 66 (43%) | 64 (47%) | 76 (51%) | 91 (49%) |
|  | Gender unknown | 0 (0.0%) | 0 (0.0%) | 0 (0.0%) | 0 (0.0%) | 1 (0.67%) | 0 (0.0%) |
| Age | Age <16 years | 6 (2.1%) | 2 (1.3%) | 2 (1.3%) | 1 (0.7%) | 3 (2.0%) | 5 (2.7%) |
|  | Age 16-59 years | 145 (51%) | 87 (56%) | 82 (53%) | 75 (55%) | 84 (56%) | 90 (48%) |
|  | Age 60-74 years | 102 (36%) | 48 (31%) | 56 (36%) | 43 (32%) | 45 (30%) | 57 (31%) |
|  | Age ≥75 years | 33 (12%) | 17 (11%) | 14 (9.1%) | 17 (13%) | 18 (12%) | 34 (18%) |
| Status | Emergency | 12 (4.2%) | 5 (3.2%) | 9 (5.8%) | 6 (4.4%) | 3 (2.0%) | 8 (4.3%) |
|  | Planned | 274 (96%) | 149 (97%) | 145 (94%) | 130 (96%) | 147 (98%) | 178 (96%) |
| Open | | | | | | | |
| Total procedures | | 1 | 6 | 7 | 14 | 10 | 11 |

Demographic data for open procedures have been censored by Public Health Scotland to prevent inadvertent disclosure. ESWL= extracorporeal shockwave lithotripsy; PCNL= percutaneous nephrolithotomy; URS= ureterorenoscopy.

Supplementary table 5. Comparison of annual activity in England

| Year | All KSD procedures | PCNL | URS | ESWL Kidney | ESWL Ureter | ESWL all | Open | TURBT | Radical cystectomy | Radical prostatectomy | Radical nephrectomy | Partial nephrectomy | All oncological resections | Benign prostatic obstruction procedure |
| --- | --- | --- | --- | --- | --- | --- | --- | --- | --- | --- | --- | --- | --- | --- |
| 2000-2001 | 35653 | 1029 | 5364 | 11484 | 3007 | 14491 | 278 | 22208 | 1337 | 1020 | 2701 | 312 | 27578 | 29611 |
| 2001-2002 | 35780 | 1117 | 5453 | 11763 | 2733 | 14496 | 218 | 21774 | 1370 | 1409 | 2656 | 306 | 27515 | 28559 |
| 2002-2003 | 36206 | 1160 | 6081 | 11885 | 2468 | 14353 | 259 | 21741 | 1434 | 1654 | 2818 | 355 | 28002 | 28220 |
| 2003-2004 | 41249 | 1245 | 6506 | 13646 | 2983 | 16629 | 240 | 21272 | 1446 | 1889 | 2720 | 414 | 27741 | 27065 |
| 2004-2005 | 42197 | 1326 | 6928 | 13973 | 2888 | 16861 | 221 | 21437 | 1447 | 2192 | 2871 | 461 | 28408 | 24863 |
| 2005-2006 | 45274 | 1441 | 7277 | 14982 | 3203 | 18185 | 186 | 23094 | 1467 | 2464 | 3180 | 469 | 30674 | 24153 |
| 2006-2007 | 47307 | 1884 | 7928 | 15564 | 3130 | 18694 | 107 | 23027 | 1540 | 2409 | 3191 | 506 | 30673 | 23814 |
| 2007-2008 | 49725 | 2230 | 8638 | 16279 | 3107 | 19386 | 85 | 23534 | 1556 | 2316 | 3226 | 605 | 31237 | 25313 |
| 2008-2009 | 55398 | 2271 | 10126 | 18684 | 2782 | 21466 | 69 | 24822 | 1621 | 2520 | 3254 | 658 | 32875 | 25755 |
| 2009-2010 | 58726 | 2587 | 11288 | 19352 | 3050 | 22402 | 47 | 24428 | 1642 | 3257 | 3082 | 800 | 33209 | 25749 |
| 2010-2011 | 61460 | 2582 | 12651 | 19926 | 3160 | 23086 | 55 | 24456 | 1766 | 3489 | 2953 | 974 | 33638 | 24811 |
| 2011-2012 | 63001 | 2778 | 13619 | 19913 | 3355 | 23268 | 68 | 25507 | 1844 | 4037 | 3337 | 1161 | 35886 | 24613 |
| 2012-2013 | 62528 | 2704 | 14818 | 19546 | 2931 | 22477 | 52 | 24927 | 1857 | 3895 | 3338 | 1332 | 35349 | 24192 |
| 2013-2014 | 65642 | 2758 | 15700 | 20263 | 3307 | 23570 | 44 | 26032 | 1891 | 4806 | 3528 | 1519 | 37776 | 24258 |
| 2014-2015 | 64612 | 2966 | 17104 | 19398 | 2858 | 22256 | 30 | 25618 | 1899 | 5302 | 3882 | 1586 | 38287 | 23472 |
| 2015-2016 | 65303 | 2935 | 18407 | 18993 | 2969 | 21962 | 37 | 25486 | 1894 | 6181 | 4006 | 1716 | 39283 | 22627 |
| 2016-2017 | 65373 | 3071 | 19856 | 18167 | 3044 | 21211 | 24 | 26016 | 1857 | 6258 | 3985 | 1817 | 39933 | 22939 |
| 2017-2018 | 64850 | 3068 | 20873 | 17329 | 3110 | 20439 | 31 | 25264 | 1863 | 6707 | 3925 | 1834 | 39593 | 22812 |
| 2018-2019 | 63851 | 3085 | 22007 | 16365 | 3002 | 19367 | 25 | 25422 | 1881 | 8391 | 3936 | 1812 | 41442 | 22644 |
| 2019-2020 | 65789 | 2988 | 21859 | 16799 | 3661 | 20460 | 22 | 25512 | 1773 | 7797 | 4001 | 1829 | 40912 | 22803 |
| 2020-2021 | 46361 | 1379 | 17968 | 9294 | 4203 | 13497 | 20 | 23208 | 1547 | 5486 | 3213 | 1531 | 34985 | 11846 |
| 2021-2022 | 63105 | 1876 | 22844 | 14519 | 4659 | 19178 | 29 | 25757 | 1806 | 6546 | 3704 | 2025 | 39838 | 18744 |
| 2022-2023 | 61920 | 1731 | 23253 | 14148 | 4309 | 18457 | 22 | 25446 | 1766 | 7559 | 3755 | 2162 | 40688 | 20487 |
| 2023-2024 | 65974 | 1692 | 25693 | 14841 | 4439 | 19280 | 29 | 27287 | 1722 | 8042 | 4085 | 2454 | 43590 | 22359 |
| 2024-2025 | 71048 | 1765 | 28860 | 15929 | 4268 | 20197 | 29 | 28418 | 1771 | 9062 | 4507 | 2683 | 46441 | 26299 |
| % change 2019/20 to 2020/21 | 30% | 54% | 18% | 45% | -15% | 34% | 9% | 9% | 13% | 30% | 20% | 16% | 14% | 48% |

Supplementary table 6. Historic trends in diagnoses and procedures

| Year | Total finished consultant episodes | Proportion of finished consultant episodes having procedural intervention | | | | | |
| --- | --- | --- | --- | --- | --- | --- | --- |
|  |  | Any intervention | ESWL (kidney) | ESWL (ureter) | URS | PCNL | Open |
| 2000 -2001 | 51035 | 0.41 | 0.23 | 0.06 | 0.11 | 0.02 | 0.01 |
| 2001 -2002 | 50161 | 0.42 | 0.23 | 0.05 | 0.11 | 0.02 | 4.35X10-3 |
| 2002 -2003 | 52249 | 0.42 | 0.23 | 0.05 | 0.12 | 0.02 | 4.96X10-3 |
| 2003 -2004 | 58944 | 0.42 | 0.23 | 0.05 | 0.11 | 0.02 | 4.07X10-3 |
| 2004 -2005 | 62914 | 0.40 | 0.22 | 0.05 | 0.11 | 0.02 | 3.51X10-3 |
| 2005 -2006 | 67817 | 0.40 | 0.22 | 0.05 | 0.11 | 0.02 | 2.74X10-3 |
| 2006 -2007 | 72237 | 0.40 | 0.22 | 0.04 | 0.11 | 0.03 | 1.48X10-3 |
| 2007 -2008 | 72957 | 0.42 | 0.22 | 0.04 | 0.12 | 0.03 | 1.17X10-3 |
| 2008 -2009 | 77870 | 0.44 | 0.24 | 0.04 | 0.13 | 0.03 | 8.87X10-4 |
| 2009 -2010 | 83050 | 0.44 | 0.23 | 0.04 | 0.14 | 0.03 | 5.66X10-4 |
| 2010 -2011 | 85154 | 0.45 | 0.23 | 0.04 | 0.15 | 0.03 | 6.40X10-4 |
| 2011 -2012 | 84323 | 0.47 | 0.24 | 0.04 | 0.16 | 0.03 | 8.07X10-4 |
| 2012 -2013 | 82206 | 0.49 | 0.24 | 0.04 | 0.18 | 0.03 | 6.33X10-4 |
| 2013 -2014 | 86054 | 0.50 | 0.24 | 0.04 | 0.19 | 0.03 | 5.19X10-4 |
| 2014 -2015 | 86742 | 0.49 | 0.22 | 0.03 | 0.20 | 0.03 | 3.46X10-4 |
| 2015 -2016 | 86619 | 0.50 | 0.22 | 0.03 | 0.21 | 0.03 | 4.28X10-4 |
| 2016 -2017 | 87610 | 0.50 | 0.21 | 0.03 | 0.23 | 0.04 | 2.75X10-4 |
| 2017 -2018 | 87347 | 0.51 | 0.20 | 0.04 | 0.24 | 0.04 | 3.56X10-4 |
| 2018 -2019 | 89177 | 0.50 | 0.18 | 0.03 | 0.25 | 0.03 | 2.81X10-4 |
| 2019 -2020 | 88632 | 0.51 | 0.19 | 0.04 | 0.25 | 0.03 | 2.43X10-4 |
| 2020 -2021 | 66049 | 0.50 | 0.14 | 0.06 | 0.27 | 0.02 | 3.04X10-4 |
| 2021 -2022 | 79412 | 0.55 | 0.18 | 0.06 | 0.29 | 0.02 | 3.66X10-4 |
| 2022 -2023 | 77152 | 0.56 | 0.18 | 0.06 | 0.30 | 0.02 | 2.86X10-4 |
| 2023 -2024 | 81083 | 0.58 | 0.18 | 0.05 | 0.32 | 0.02 | 3.54X10-4 |
| 2024 -2025 | 84130 | 0.60 | 0.19 | 0.05 | 0.34 | 0.02 | 3.48X10-4 |

Supplementary table 7. Projected activity for kidney stone disease from 2025-2035

| Year | Finished consultant episodes  (95% CI) | ESWL procedures  (95% CI) | URS procedures  (95% CI) | PCNL procedures  (95% CI) | Open procedures  (95% CI) |
| --- | --- | --- | --- | --- | --- |
| 2025-26 | 94862 (85928-104724) | 21490 (19466-23724) | 32582 (29514-35969) | 2823 (2557-3117) | 8 (7-9) |
| 2026-27 | 96620 (87024-107273) | 21678 (19525-24068) | 34684 (31240-38509) | 2893 (2606-3212) | 7 (6-8) |
| 2027-28 | 98410 (88125-109896) | 21868 (19582-24420) | 36882 (33028-41187) | 2964 (2655-3310) | 6 (5-7) |
| 2028-29 | 100234 (89232-112592) | 22059 (19638-24779) | 39178 (34877-44008) | 3038 (2704-3412) | 5 (5-6) |
| 2029-30 | 102091 (90346-115362) | 22253 (19693-25146) | 41570 (36787-46974) | 3112 (2754-3517) | 4 (4-5) |
| 2030-31 | 103983 (91469-118208) | 22448 (19747-25519) | 44058 (38756-50086) | 3189 (2805-3626) | 4 (3-4) |
| 2031-32 | 105910 (92601-121131) | 22645 (19799-25900) | 46644 (40782-53347) | 3268 (2857-3738) | 3 (3-4) |
| 2032-33 | 107872 (93743-124132) | 22844 (19852-26287) | 49324 (42863-56758) | 3348 (2910-3853) | 3 (2-3) |
| 2033-34 | 109871 (94895-127212) | 23044 (19903-26681) | 52098 (44997-60321) | 3431 (2963-3973) | 2 (2-3) |
| 2034-35 | 111907 (96057-130373) | 23247 (19954-27083) | 54966 (47180-64035) | 3516 (3018-4096) | 2 (2-2) |

95% CI= 95% confidence interval; ESWL= extracorporeal shockwave lithotripsy; PCNL= percutaneous nephrolithotomy; URS= ureterorenoscopy

Analyses assume that trends in number episodes attributable to diagnostic codes associated with kidney stone disease from 1998-1999 to 2024-2025 continue

Supplementary table 8. Projected procedural expenditure on kidney stone disease from 2025-2035

| Year | Procedure | Projected likely cost  (£ millions) | Projected minimum cost  (£ millions) | Projected maximum cost  (£ millions) |
| --- | --- | --- | --- | --- |
| 2025-26 | All diagnoses | £136.05 | £90.71 | £198.24 |
|  | ESWL | £19.20 | £17.39 | £21.20 |
|  | URS | £92.91 | £84.16 | £102.57 |
|  | PCNL | £16.06 | £14.55 | £17.73 |
|  | Open | £0.06 | £0.05 | £0.06 |
| 2026-27 | All diagnoses | £138.67 | £91.39 | £203.91 |
|  | ESWL | £18.85 | £16.98 | £20.93 |
|  | URS | £96.27 | £86.71 | £106.88 |
|  | PCNL | £16.02 | £14.43 | £17.79 |
|  | Open | £0.05 | £0.04 | £0.05 |
| 2027-28 | All diagnoses | £146.57 | £95.50 | £217.49 |
|  | ESWL | £19.20 | £17.19 | £21.44 |
|  | URS | £103.34 | £92.54 | £115.40 |
|  | PCNL | £16.57 | £14.84 | £18.51 |
|  | Open | £0.04 | £0.04 | £0.05 |
| 2028-29 | All diagnoses | £158.03 | £101.79 | £236.61 |
|  | ESWL | £19.95 | £17.76 | £22.41 |
|  | URS | £113.07 | £100.66 | £127.01 |
|  | PCNL | £17.49 | £15.57 | £19.65 |
|  | Open | £0.04 | £0.03 | £0.04 |
| 2029-30 | All diagnoses | £170.33 | £108.48 | £257.31 |
|  | ESWL | £20.73 | £18.34 | £23.42 |
|  | URS | £123.57 | £109.35 | £139.63 |
|  | PCNL | £18.46 | £16.34 | £20.86 |
|  | Open | £0.03 | £0.03 | £0.04 |
| 2030-31 | All diagnoses | £183.52 | £115.58 | £279.71 |
|  | ESWL | £21.53 | £18.94 | £24.48 |
|  | URS | £134.90 | £118.66 | £153.35 |
|  | PCNL | £19.48 | £17.14 | £22.15 |
|  | Open | £0.03 | £0.03 | £0.03 |
| 2031-32 | All diagnoses | £197.65 | £123.12 | £303.90 |
|  | ESWL | £22.37 | £19.56 | £25.59 |
|  | URS | £147.09 | £128.61 | £168.23 |
|  | PCNL | £20.56 | £17.98 | £23.52 |
|  | Open | £0.03 | £0.02 | £0.03 |
| 2032-33 | All diagnoses | £212.77 | £131.11 | £330.02 |
|  | ESWL | £23.25 | £20.20 | £26.75 |
|  | URS | £160.21 | £139.23 | £184.36 |
|  | PCNL | £21.70 | £18.86 | £24.97 |
|  | Open | £0.02 | £0.02 | £0.03 |
| 2033-34 | All diagnoses | £228.94 | £139.57 | £358.18 |
|  | ESWL | £24.16 | £20.86 | £27.97 |
|  | URS | £174.30 | £150.54 | £201.81 |
|  | PCNL | £22.90 | £19.78 | £26.52 |
|  | Open | £0.02 | £0.02 | £0.02 |
| 2034-35 | All diagnoses | £246.21 | £148.52 | £388.50 |
|  | ESWL | £25.10 | £21.54 | £29.24 |
|  | URS | £189.41 | £162.58 | £220.67 |
|  | PCNL | £24.17 | £20.75 | £28.16 |
|  | Open | £0.02 | £0.02 | £0.02 |
